# Ultracongruent insert design is a safe alternative to posterior cruciate-substituting total knee arthroplasty: 5-year results of a randomized controlled trial

**DOI:** 10.1007/s00167-021-06545-4

**Published:** 2021-04-11

**Authors:** Jörg Lützner, Franziska Beyer, Cornelia Lützner, Roman Riedel, Eric Tille

**Affiliations:** University Center of Orthopaedic, Trauma and Plastic Surgery, University Hospital Carl Gustav Carus, TU Dresden, Fetscherstr. 74, 01307 Dresden, Germany

**Keywords:** TKA, TKR, Anterior stabilized, Posterior stabilized, Ultracongruent insert, Deep dished insert, Stability, Range of motion, Results, Patient-reported outcome

## Abstract

**Purpose:**

If substitution of the posterior cruciate ligament in total knee arthroplasty is necessary, there are two options available: posterior stabilized (PS) design with a post-cam mechanism or anterior-lipped ultracongruent (UC) inserts. UC inserts have the advantage that no femoral box is necessary and a standard femoral component can be used. The aim of this study was to compare the range of motion (ROM) and patient-reported outcome (PRO) after UC and PS fixed-bearing TKA. Better ROM in PS TKA and no difference in PRO between both designs was hypothesized.

**Methods:**

A randomized controlled trial with 127 patients receiving a fixed-bearing UC or PS design of the same knee system was performed. Nine patients died and there were four revision surgeries. 107 patients completed the 5-year follow-up. Patient-reported outcome was assessed. Patellofemoral problems were evaluated using selected applicable questions of the Oxford Knee Score (getting up from a table, kneeling, climbing stairs).

**Results:**

Surgical time was 10 min shorter in the UC group (*p* < 0.001). After 5 years, both groups demonstrated good knee function and health-related quality of life without significant differences between the groups. Both groups demonstrated a high satisfaction score and the majority of patients would undergo this surgery again. Patellofemoral problems were recognized more frequently in the PS group (*p* = 0.025).

**Conclusion:**

Both designs demonstrated similar good results after 5 years. Stabilization with an anterior-lipped UC insert can be considered a safe alternative to the well-established PS design if cruciate substitution is necessary.

**Level of evidence:**

1.

## Introduction

The decision to sacrifice or preserve the posterior cruciate ligament (PCL) in total knee arthroplasty (TKA) mainly depends on surgeon preference. Despite better kinematic performance of PS TKA, no difference in outcome has been demonstrated between retention and sacrifice of the PCL except for slightly better knee flexion in PS TKA [6]. However, if the PCL is insufficient, absent, or needs to be resected, the substitution of the PCL is necessary [4, 20, 26]. In these situations, there are two options for substitution of the PCL: posterior-stabilized (PS) implants with a box and cam mechanism or anterior-lipped ultracongruent (UC) inserts. The PS design needs additional bone resection of the femur for preparation of the box which increases surgical time and fracture risk [20]. Furthermore, there is additional cam-mechanism polyethylene wear, a risk of dislocation [22] and the patella clunk syndrome may occur [16]. The use of a highly conforming ultracongruent (UC) insert with a standard femoral component is a simple alternative to the established PS TKA design [15, 20]. However, the UC design has potential disadvantages. While some studies demonstrated a reduced range of motion (ROM) for UC compared to PS design [24, 32], other studies found similar ROM for both designs [18, 19]. Some studies found reduced axial rotation in UC compared to PS TKA [11, 18, 25], which might result in increased long-term polyethylene (PE) wear in a highly congruent fixed-bearing design. For many TKA brands, anterior-lipped UC inserts are available. Despite the regular use of these UC inserts, there is only limited evidence about mid- or long-term outcome. Only few studies report 5- or 10-year results, none with an appropriate control group [3, 10, 18].

## Purpose and hypothesis

The aim of this study was to compare the range of motion (ROM) and patient-reported outcome (PRO) after UC and PS fixed-bearing TKA. Better ROM in PS TKA and no difference in PRO between both designs was hypothesized.

## Materials and methods

**Fig. 1:**
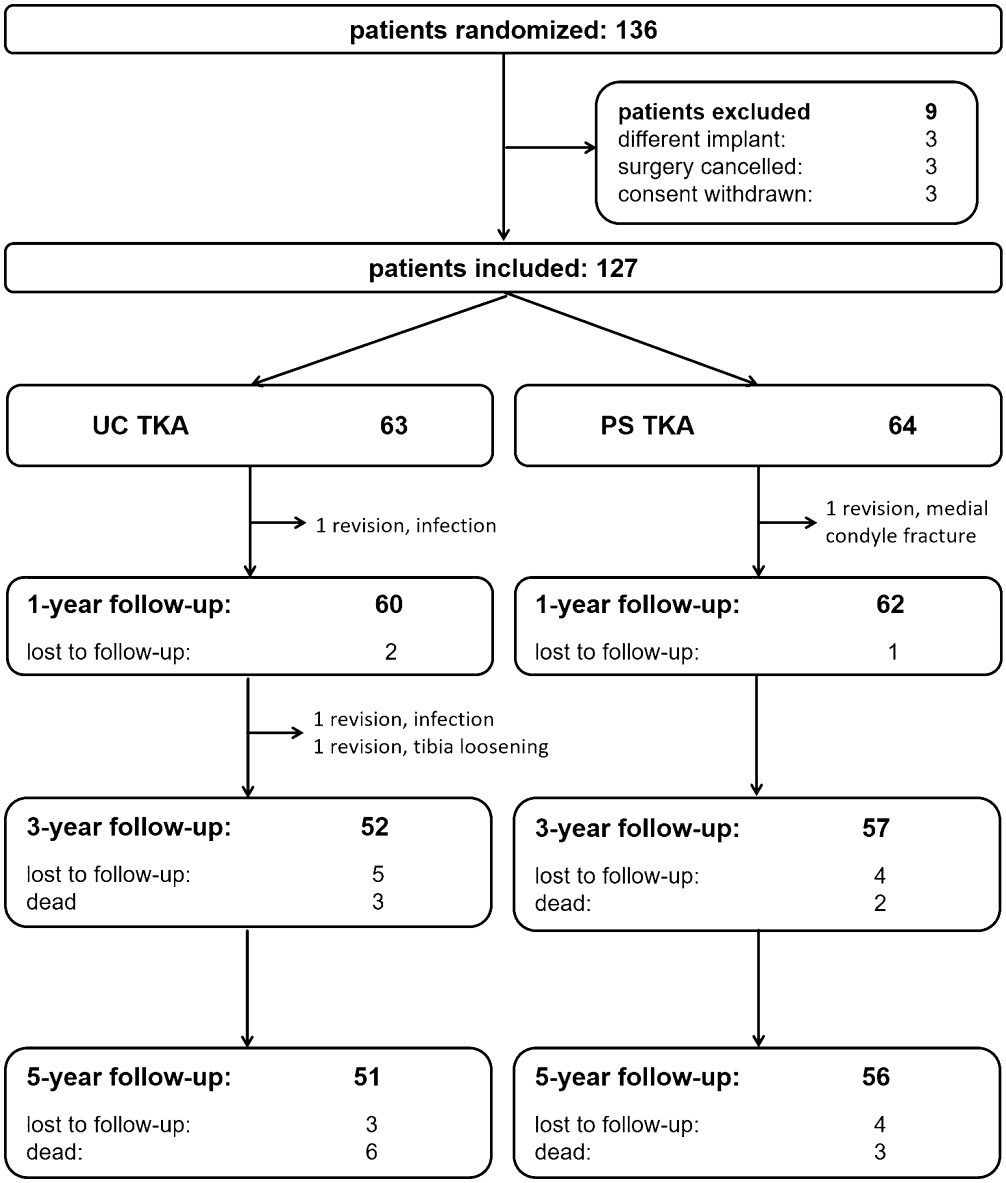
Flow chart of study patients

In a randomized controlled trial [23], all patients scheduled for a condylar TKA were screened. Patients with the need for a higher constraint TKA (condylar constraint or rotating hinge TKA) were not included. A total of 127 patients were included. Patients were interviewed by a trained study nurse before surgery, 3 months, as well as 1, 3 and 5 years after surgery. Nine patients died, four patients required revision surgery and seven patients were lost to follow-up, resulting in 51 patients with an UC and 56 patients with a PS design at the final follow-up (Fig. 1). ROM was measured with a goniometer preoperatively and at each follow-up. Knee Function (Oxford Knee Score, OKS [28]), physical activity (University of Los Angeles (UCLA) activity scale [2]) and health-related quality of life (SF36 [9]) were obtained. At the final follow-up, patients were asked how satisfied they were with the outcome of TKA. It was assessed on a visual analogue scale from 0 (very dissatisfied) to 10 (very satisfied) [8]. Additionally, patients were asked if they would undergo TKA again, if necessary. Patients could choose between five possible answers: definitely yes, possibly yes, not sure, probably not, certainly not [13]. Sociodemographic data, operative details and revisions were assessed for all patients. Surgical time was measured from skin incision to wound closure.

The surgeries were performed by two experienced arthroplasty surgeons. All patients received a cemented fixed-bearing TKA (Columbus, B. Braun Aesculap, Tuttlingen, Germany) without patellar resurfacing. Both cruciate ligaments were resected. A gap-balancing technique was used with the help of a navigation system (Orthopilot, Software TKR v4.3, B. Braun Aesculap, Tuttlingen, Germany). Tibial and femoral bone cuts, sizing of the femoral component and soft-tissue balancing were performed as indicated by the navigation system. For the UC design a standard femoral component was used, for the PS design additional preparation of the box was performed.

The study was registered at ClinicalTrials.gov (NCT04679857). All patients signed an informed consent.

### Statistical analysis

The sample size was calculated to detect a minimum clinically relevant difference of 5° of knee flexion between the two implant designs. With a power of 80%, a significance level of 0.05 and an estimated standard deviation of 10°, a number of 64 patients in each group were necessary.

Patient characteristics, data from TKA surgery, comorbidities (ASA grade) and adverse events were recorded prospectively, assembled into a database together with the questionnaires and analysed using SPSS^®^ software (release 26 for Windows^®^). The acquired data did not show a normal distribution (Kolmogorov–Smirnov test) and therefore reported as median, 25th and 75th percentiles for continuous values and absolute and relative frequencies for categorical values, respectively. To compare study groups, Mann–Whitney *U* test for continuous values and chi-square test for categorical values were used.

## Results

There were no differences between the study groups 5 years postoperatively regarding age, gender, BMI and comorbidities. Surgical time was 10 min longer in the PS group (*p* < 0.001, Table 1).

**Table 1:** Patient characteristics, given as median (25th percentile, 75th percentile) and relative frequencies

Patient characteristics	UC TKA	PS TKA	*p* value
Age at surgery (years)	71 (62;77)	72 (62;77)	n.s
Gender (female)	72.5%	64.3%	n.s
BMI (kg/m^2^)	31.1 (27.9; 35.4)	29.4 (27.6; 34.1)	n.s
ASA grade			
1 and 2 3 and 4	47.1% 52.9%	44.6% 55.4%	n.s
Surgical time (min)	86 (80; 94)	96 (88; 101)	< 0.001

Knee flexion increased from a median 105° in the UC group and 100° in the PS group before surgery to 115° in both groups at the final follow-up. Knee function (OKS), activity (UCLA activity scale) and health-related quality of life (SF36) demonstrated significant improvement in both groups without significant differences (Table 2).

**Table 2:** PRO before surgery, and 1, 3 and 5 years after surgery, given as median values (25th percentile, 75th percentile)

Score	UC TKA	PS TKA	*p* value
Oxford Knee Score (max. 48 points)			
Before surgery	20 (17, 25)	22 (18, 25)	0.370
1-year follow-up	42 (36, 45)	37 (29, 43)	0.004
3-year follow-up	42 (36, 46)	38 (33, 42)	0.012
5-year follow-up	42 (37, 45)	41 (27, 44)	0.101
Improvement	19 (14, 26)	17 (10, 22)	0.034
OKS pain component (max. 100 points)			
Before surgery	39 (29, 54)	41 (29, 50)	0.795
1-year follow-up	93 (79, 100)	86 (64, 93)	0.008
3-year follow-up	93 (82, 100)	82 (75, 96)	0.037
5-year follow-up	93 (82, 100)	93 (68, 96)	0.335
Improvement	46 (36, 61)	46 (32, 96)	0.314
OKS function component (max. 100 points)			
Before surgery	45 (35, 55)	50 (38, 60)	0.123
1-year follow-up	80 (65, 90)	70 (55, 83)	0.009
3-year follow-up	80 (65, 95)	70 (60, 80)	0.049
5-year follow-up	80 (60, 95)	70 (48, 85)	0.069
Improvement	30 (20, 40)	15 (5, 35)	0.004
UCLA activity scale (max. 10 points)			
Before surgery	4 (3, 5)	4 (3, 5)	0.954
1-year follow-up	5 (3, 6)	5 (3, 6)	0.807
3-year follow-up	5 (3, 6)	5 (3, 6)	0.475
5-year follow-up	4 (4, 5)	4 (3, 6)	0.511
Improvement	1 (-1, 2)	1 (0, 2)	0.639
SF36 physical scale^a^			
Before surgery	23.8 (18.9, 31.8)	25.6 (19.4, 31.8)	0.635
1-year follow-up	48 (33.6, 53.5)	39.6 (29, 50.3)	0.045
3-year follow-up	38.5 (25.7, 52.3)	37.2 (28.8, 46)	0.737
5-year follow-up	42.3 (27.4, 52.3)	37.9 (24.5, 50.1)	0.275
Improvement	14.2 (8.2, 24.3)	10.6 (2, 19.4)	0.112
SF36 mental scale^b^			
Before surgery	57.8 (46, 65.8)	56.4 (46.9, 63.9)	0.585
1-year follow-up	55.7 (43.9, 59.6)	53.2 (43.7, 58.1)	0.450
3-year follow-up	54.7 (43.7, 59.4)	53.4 (46.8, 59.9)	0.684
5-year follow-up	56.5 (49.5, 62)	54.5 (48.4, 60.3)	0.388
Improvement	-3.1 (-9.7, 6.3)	-3.1 (-8.4, 5.2)	0.820
Satisfaction with TKA (max. 10.0)			
5-year follow-up	9.0 (7.5, 10.0)	8.8 (7.3, 9.5)	0.269

^a^Norm values for German population, age 61–70 years: 48.1 (38.0; 53.7), age > 70 years: 40.8 (31.0; 50.1)

^b^Norm values for German population, age 61–70 years: 55.1 (50.0; 58.4), age > 70 years: 53.6 (48.9; 58.9)

**Fig. 2:**
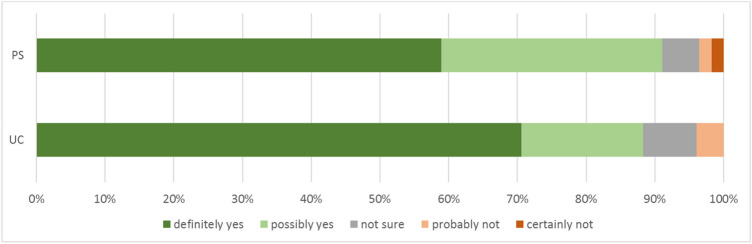
Willingness to undergo the surgery again (if necessary)

The satisfaction score was high in both groups. The majority of patients (89.7%) indicated that they would undergo TKA surgery again, if necessary (Fig. 2).

**Fig. 3:**
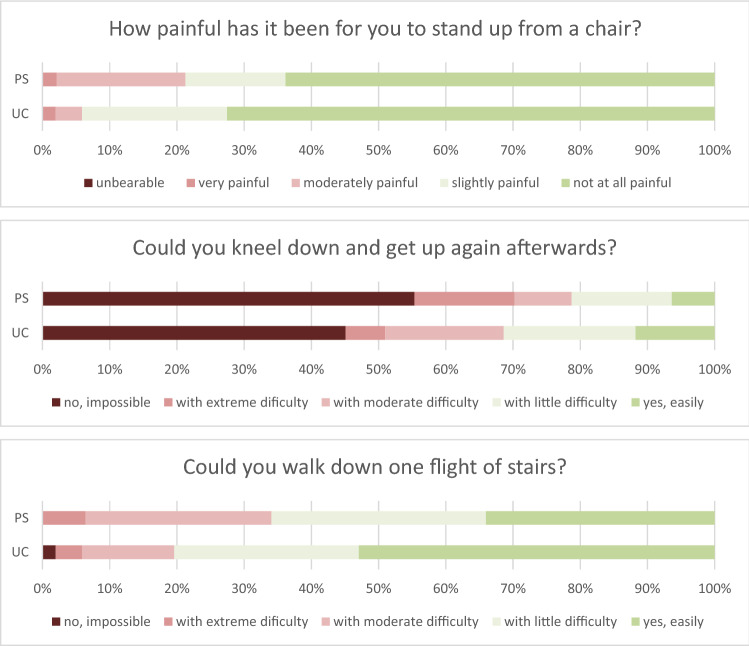
Questions from the OKS which are linked to patellofemoral problems

Looking at the items in the OKS regarding activities with a high patellofemoral load, there were better results for the UC design for getting up from a table (*p* = 0.025). Patients in both groups had the greatest difficulties in kneeling, which was impossible or extremely difficult in 51% in the UC group and 70% in the PS group (*p* = 0.052, Fig. 3).

Four patients required revision surgery until the 5-year follow-up. One patient in the PS group experienced a medial condyle fracture 3 months after surgery, which might have been missed during the initial surgery. Two patients in the UC group had a deep infection and staged revision TKA was performed. One patient in the UC group required partial revision of the tibial component due to aseptic loosening 29 months after surgery.

## Discussion

The most important finding of this study was that there was no difference in ROM, knee function, physical activity, health-related QoL or satisfaction in fixed-bearing TKA after 5 years using either UC or PS design.

The use of cruciate retaining or substituting TKA is a matter of surgeon philosophy. Despite better reproduction of the femoral rollback during flexion and therefore more natural knee kinematics in PS TKA, no conclusive evidence supports the superiority of one design above the other in clinical practice [5, 27]. Regardless of surgeon preferences for one design, there are certain situations in which substitution of the PCL is obligatory: if the PCL is damaged, absent or needs to be resected. Bae et al. have analysed a series of 920 patients planned for a cruciate-retaining TKA [4]. In 83 knees (9%) an intraoperative conversion to a PS design was necessary. In case of unplanned need for PCL substitution, the UC design offers advantages compared to the traditional PS design: no additional bone resection is necessary and a standard femoral component can be used making additional implant storage in the OR obsolete.

Kinematic studies demonstrated better ROM, less anteroposterior laxity and more posterior femoral rollback of PS compared to UC TKA [5, 11, 17]. These kinematic aspects seem to have no impact on clinical and patient-reported outcome in either approach of TKA. Kim et al. compared UC and PS TKA in the same patient and found no difference in side preference, satisfaction or joint perception despite kinematic advantages of the PS design [17]. Akti et al. investigated isokinetic performance and found no difference between UC and PS TKA [1]. Several studies compared short-time results between UC and PS TKA [1, 7, 17, 18, 20, 23, 24, 29, 30]. In these studies, different TKA designs were investigated and different patient-reported outcome measures were used, but all studies reported similar results for UC and PS TKA. There were no differences in revision rates or KOOS-JR scores between PS and UC TKA after mean 43 months follow-up in a large consecutive series of 5970 patients by Yacovelli et al. [33]. Only one study reported long-term results including fixed-bearing (*n* = 38) and mobile-bearing UC TKA (*n* = 199) [3]. There was no difference between all implant designs in survival after 10 years. Unfortunately, UC TKA is not clearly reported separately in the major arthroplasty registries. Looking at survivorship in the registries, there are generally higher revision rates for higher constraint TKA. In the Australian Arthroplasty Registry, high-volume surgeons preferring PS TKA had a 45% higher risk of revision at 13 years compared to surgeons preferring minimally stabilised TKA [31]. Two recent reviews [5, 27] summarized available evidence and found no difference in clinical outcome between UC and PS TKA which is consistent with the results of the present study. After reviewing kinematic and clinical studies as well as registry data, Meneghini et al. [27] concluded that a post-cam mechanism is no longer necessary in modern primary TKA. These results suggest that UC TKA is a safe option.

While there was no overall difference between both groups, activities with high patellofemoral load (getting up, kneeling, climbing stairs) appeared to be more troublesome for patients in the PS group. The majority of patients in both groups had severe difficulties with or were unable to kneel. In vitro studies demonstrated that changes in patellofemoral kinematics and patellofemoral pressure increase after TKA depending on TKA design. This may have an influence on patellofemoral pain [12, 21]. In vitro measurements demonstrated higher patellofemoral pressure in UC TKA compared with the PS design [14]. This is in contrast to the present study and might be explained by differences in the design of the anterior femur between implant brands and whether patellar resurfacing was performed or not. In the present study, no patellar resurfacing was performed. The patellar clunk syndrome has been described as a result of a large box design in PS TKA [16]. Consequently, the box design and the design of the anterior femur has been changed to become more “patella-friendly”. Consequently, this does not seem to be an issue in modern PS design any longer.

This study has some limitations. Not all eligible patients could be included and few patients were lost to follow-up, therefore a risk of selection bias exists. This study was performed using a specific fixed-bearing TKA design. Results may therefore not be applicable to other TKA designs. Patellofemoral problems were assessed using the questions from the OKS. No specific validated score for patellofemoral pain was used. Despite these limitations, this study provides detailed information on mid-term outcome of UC and PS TKA.

## Conclusion

This study demonstrated no differences in ROM and PRO between fixed-bearing UC and PS TKA during mid-term follow-up. UC TKA can be considered a safe alternative to the well-established PS design in PCL substituting TKA. For surgeons who do not always substitute the PCL, this may be an advantage.
